# 

**Published:** 1891-09

**Authors:** 


### BOOKS AND PAMPHLETS RECEIVED.

*American Veterinary College*, New York. — Seventeenth Annual Announcement, Session 1891-1892. Catalogue, 1890-1891. List of Graduates to 1891, and Report of Hospital Department, 1890-'91. This announcement shows a steady augmentation in the facilities of instruction, both theoretical and practical, which are offered to the students, and in the number of students in attendance. There has been a decided increase of the latter in the last few years. The next Session commences October 5th, 1891.

*Ontario Veterinary College*, Toronto Canada.—Annual Announcement, Session 1891-'92, also names of the graduates who hold the Diploma of the Council of the Agriculture and Arts Association. The new buildings are described, and are represented in several illustrations. A list of the graduates shows a large number from the United States. The next Session commences October 21st, 1891.

Bulletin Société Linneenne du Nord de la Erance. Amienes

